# Protection and Active Decontamination of Dairy Cattle Heifers against Lipophilic Toxins (PCBs) from Diet

**DOI:** 10.3390/toxics9040080

**Published:** 2021-04-08

**Authors:** Alexander Sotnichenko, Elena Tsis, Magomed Chabaev, Vasily Duborezov, Alexander Kochetkov, Roman Nekrasov, Victor Okhanov

**Affiliations:** 1 Research and Production Center “Fox and Co”, LLC, 117149 Moscow, Russia; company@fox-rpc.com; 2 L.K. Ernst Federal Research Center for Animal Husbandry, Podolsk Urban District, Dubrovitsy Settlement, 142132 Moscow, Russia; eyutsis@vij.ru (E.T.); mgchabaev@vij.ru (M.C.); korma10@yandex.ru (V.D.); rvnekrasov@vij.ru (R.N.); 3 Scientific and Production Association “Typhoon”, Institute for Environmental Monitoring, Kaluga Region, 249038 Obninsk, Russia; kochetkov@rpatyphoon.ru

**Keywords:** dairy heifers, weight gain, PCBs, adsorbent, POPSH, decontamination

## Abstract

We studied the effects of a hydrophobized reversed-phase feed adsorbent in the form of a polyoctylated polysilicate hydrogel (POPSH) on productivity indicators, metabolic adaptation, and on the level of polychlorinated biphenyls (PCBs) in the blood of growing Holsteinized black-and-white heifers during the transition period. Two groups of two-month-old heifers of 20 head each were used. The experimental group received POPSH in addition to the main diet. The use of the adsorbent led to an increase in daily weight gain by 19.9% and to a decrease in the total concentration of PCB congeners found in whole blood by 40%. The greatest decrease in concentration (35–52%) was observed for tetra-, penta- and hexachlorobiphenyls. These results demonstrate the possibilities of effective protection of calves from lipophilic toxins of feed and their active decontamination.

## 1. Introduction

Dairy calves are at high risk of morbidity and mortality, especially during milk feeding, milk replacer feeding and in the first weeks after switching to the main diet. In this regard, when rearing young stock for herd maintenance, most attention is paid to the quantity and quality of feeds used and their composition, especially during the transition period from a dairy diet to a diet with solid feed, when effective cicatricial digestion begins to form [1]. At the current level of environmental pollution, the safety and quality of feed are of the utmost importance in this period [2]. Unfortunately, the problems of obtaining biologically valuable and harmless livestock products remain unresolved, both in scientific-theoretical and practical terms. Research results show that animal husbandry suffers serious economic losses due to a decrease in the productivity and reproduction of farm animals caused by poor quality feed [1,2,3,4].

Feed used in animal husbandry almost always contains extraneous toxic impurities [2,4,5], the presence of which cannot but affect the health of productive animals, especially young ones. Among such impurities, ubiquitous in plant feed, various authors primarily name mycotoxins [4,6]. In addition to mycotoxins, feed for dairy and beef cattle, in practice, always contains two more groups of toxic compounds which, as a result of natural phenomena and human activities, are widespread in the environment of any region. These are polyaromatic hydrocarbons (PAHs) and persistent organic pollutants (POPs) [2,5,7,8].

Without exception, all PAHs, POPs in addition to 45% of known mycotoxins are lipophilic substances [9], prone to bioaccumulation [10,11]. It has been established that PAHs and POPs are capable of transferring from feed to eggs [12], meat and milk [2,5,13,14,15] in different quantities. Due to their high metabolic stability, this is especially typical of POPs, including polychlorinated biphenyls (PCBs) [2,5,7,8,12,13].

At present, it is not possible to assess which groups of toxins from feed cause the greatest damage to animal husbandry: mycotoxins, PAHs or POPs. The situation is aggravated by the fact that feed, depending on the geographic location of the area in which it is harvested, contains mixtures of tens or even hundreds of different toxic impurities in various, frequently changing concentrations and ratios. For some types of feed and localities, the greatest contribution to toxicity can be made by mycotoxins [4]; in other conditions, the influence of PAHs or POPs prevails [2,5,7], or all of these toxins act together. The influence of the combined intake of these complex and toxic mixtures into the body of an animal, even in low concentrations, and the diagnostic signs of their negative impact on the functional systems of the body are still poorly studied. For this reason, the search for effective preventive measures to reduce the toxic load from contaminated feed on the physiological resistance of animals is an urgent task for the production and use of feed in animal husbandry. 

To reduce the level of contamination, feed adsorbents have been used for a long time and often successfully [16,17,18]. Most feed adsorbents are effectively used in poultry and pig breeding in the presence of polar and moderately polar mycotoxins in feed, but often their use to remove lipophilic toxins does not lead to a positive result, even in vitro [19]. This especially applies to highly lipophilic mycotoxins and toxins such as PCBs. For instance, it was shown that the use of three polar aluminosilicate adsorbents from different manufacturers did not significantly affect the degree of transfer of dioxin and PCBs from feed to eggs by laying hens, although the authors reported some observed tendency towards a decrease in transfer [12].

The main sources of intake of polychlorinated dibenzodioxins, dibenzofurans, and biphenyls in the body of an average US citizen have been shown to be represented by animal products (meat, fish, eggs, milk and dairy products) [20]. In accordance with national food traditions, the largest contribution to the accumulation in the human body of these ecotoxicants from food is made by meat and dairy products, which are the output of cattle breeding, as the authors of the study pointed out.

It is not necessary, within the framework of this article, to describe the pathologies caused by the action on the human body of polychlorinated dibenzodioxins (PCDDs), dibenzofurans (PCDFs) and biphenyls, even at low concentrations. These issues are discussed in hundreds of scientific articles and reviews, but it is important to emphasize that urgent measures are needed to reduce the intake of these toxicants from food. Consequently, it is proposed that consideration should be given to the use of a non-polar adsorbent as a means of reducing the concentration of PCBs in food of animal origin.

We have previously shown that POPSH effectively protects broiler chickens [18], weaned piglets [21] and lactating cows [22] from toxins contained in feed. The effectiveness of its action was expressed in chickens in an increase in their productivity and survival rate; in piglets, in the normalization of metabolic processes, an increase in productivity and indicators of immune status; and in lactating cows, in an increase in productivity and a decrease in the somatic cells count (SCC) in collected milk. We have also shown that POPSH is superior to feed adsorbents based on aluminosilicates or yeast cell walls and activated carbon in the ability to bind lipophilic substances such as mycotoxin (zearalenone) or PAH (naphthalene), and it has also shown the ability to reduce the concentration of certain organochlorine pesticides (OCPs) in the milk of lactating cows [9]. Taking into account the dominant contribution of the products of beef and dairy cattle breeding to the accumulation of POPs in the human body [20], we decided to study the effectiveness of the prophylactic use of one of the non-polar reversed-phase adsorbents described earlier [23], specifically POPSH, to investigate the accumulation of PCBs from feed in the body of growing heifers when they are transferred to a solid main diet. We also studied the effect of POPSH on the main biochemical parameters of blood serum, the dynamics of weight gain, and the possibility of decontamination of animals from accumulated PCBs.

## 2. Materials and Methods

### 2.1. Material

Feed additive based on POPSH is designed to remove mycotoxins, PAHs and POPs from the feed mass in the oral cavity and in the digestive tract of animals. The adsorbent is produced under the “Alvisorb” trademark. Manufacturer: Research and Production Center, “Fox and Co”, LLC (Moscow, Russia).

### 2.2. Research Object

An experiment lasting 150 days was carried out from February to August 2018 on the basis of the Naro-Osanovsky Breeding Farm JSC (Moscow region, Odintsovo district). Forty clinically healthy Holsteinized black-and-white heifers at the age of 2 months were divided into 2 paired groups, control (C) and experimental (E), taking into account age and body weight (58.3 ± 2.65 days/67.21 ± 2.42 kg and 56.5 ± 2.42 days/67.55 ± 2.59 kg) respectively.

The studies were carried out in accordance with the requirements of the European Convention for the Protection of Vertebrate Animals used for Experimental and Other Scientific Purposes (ETS No. 123, Strasbourg, 1986). The study protocol was approved by the Bioethics Commission of the Ernst Federal Research Center for Livestock (Minutes No. 2018-02/1, dated 3 February 2018).

The industrial premises in which the research was carried out were equipped with everything necessary for organizing maintenance, feeding and drinking. Newborn calves, after drying and the first drinking of colostrum, were placed in individual dispensary cages, where they remained until 21 days of age. All this time, the calves were not allowed to the udder and received colostrum and milk from the bottle. They were then transferred to boxes for group keeping, 5 head each.

The experiment was carried out in accordance with the Methodology for Scientific Research in Livestock [24]. During the period of the experiment, the rations of the experimental animals were used in accordance with the current recommendations on the daily requirements for nutrients and energy [25].

The main diet consisted of traditional homemade feeds in the form of colostrum, pasteurized milk, succulent feed (Timothy-clover haylage, corn silage), roughage (hay of perennial grasses) and purchased feed in the form of a prestarter brand, “KK-62”, compound feed brand, “KK-64 “(AgroVitex, LLC, Moscow, Russia) and whole milk replacer (WMR) brand “Logas Milk Standard”, (Voronovsky plant of regenerated milk, Moscow, Russia).

Up to the age of 60 days (the beginning of the experiment) and in the first 30 days after its beginning, colostrum and pasteurized milk from the same dairy farm were used to feed the calves. From the 30th day of the experiment, the calves had access to milk, hay, haylage and prestarter. From the 60th day, the calves had access to the silage, and from the 70th day, the milk was replaced with WMR. From the 90th day, the prestarter was replaced with the KK-64 compound feed, and by the 120th day the calves were weaned from WMR and completely transferred to a diet with solid feed. Until the end of the experiment (120–150 days), the calves received a basic diet consisting of hay, haylage, silage, and compound feed. Samples from this diet were analyzed for PCB content.

Characterizing the structure of the solid part of the diet, it should be noted that its saturation with concentrates was more than 50% calculated on dry matter (Table 1).

During the study on the farm, the calves were fed twice a day: 50% of the daily ration during the morning feeding and the remaining 50% in the evening. In the experimental group, the main diet was supplemented with POPSH in the amount of 0.25 g/kg of live weight in the morning feeding for five consecutive days, with a two-day break.

The heifers were weighed individually on a monthly basis from the beginning of the experiment until its completion to determine the gross average daily gain in live weight. Specific feed costs were estimated based on the consumption of digestible protein per unit of live weight gain.

### 2.3. Blood Sampling and Biochemical Studies

Blood samples were taken from animals (N = 20, n = 5) at the beginning and at the end of the experiment from the tail vein into Improvacuter vacuum tubes (Arkray, Japan). The blood samples were delivered on melting ice to the laboratory, and subjected to centrifugation (3000 rpm) for 20 min, before further storage of the separated serum at −20 °C until analysis.

Concentrations of calcium (Ca), phosphorus (P), magnesium (Mg), total bilirubin (BIL), cholesterol (Chol), glucose (Glu), triglycerides (TG), total protein (TP) and the activity of aspartate aminotransferase (AST), alanine aminotransferase (ALT) and alkaline phosphatase (ALP) were determined in the serum according to a standard protocol on a Chem Well automatic biochemical analyzer (Awareness Technology, Palm City, FL, USA).

### 2.4. Determination of Polychlorinated Biphenyls

At the end of the study, 5 mL blood samples were taken from animals of both groups (N = 19 and n = 5 in each) to determine the concentration of PCB congeners. The samples were combined into averaged samples for each group of animals, frozen, and stored at −20 °C until analysis. The mass fraction of PCBs in the samples (feed, milk, WMR) was determined according to the interstate standard method for the determination of PCBs in feed and food products [26]. Blood samples were analyzed according to the same method, but the preparation of samples before analysis was carried out according to the method described for the determination of PCBs in blood serum [27]. The results were expressed in nanograms per 1 g of the lipid fraction of the sample (milk, WMR and blood) or in nanograms per 1 g of dry matter (solid feed). To characterize PCBs, we used the values of the partition coefficients in the octanol/water system (Log Pow) given in [28]. To characterize PCDDs and PCDFs, we used the values of the partition coefficients in the octanol/water system (Log Pow) given in [29].

### 2.5. Statistical Analysis

The main statistical values were calculated, and the significance of the differences was determined by comparing the results obtained using the one-way analysis of variance (ANOVA) in the STATISTICA 10 program (StatSoft, Moscow, Russia). In this way, the following values were calculated: arithmetic mean (M), root-mean-square error (m) and observed significance level (P).

## 3. Results and Discussion

### 3.1. General Health Status of Heifers

Throughout the experiment, the health of the heifers in both groups was satisfactory. The cases in this study were recorded based on the veterinary diagnosis report (Table 2).

Three animals from each group had gastrointestinal diseases, characterized by varying severity from short-term mild indigestion to severe diarrhea. One calf in the control group was reported to have a respiratory disease and was removed from the experiment. No other cases of pathology were found.

The use of POPSH helped to reduce duration of diarrhea in the experimental group by 2–3 days in comparison with the control group. The heifers from the experimental group were characterized by a more solid consistency of excrement compared to the control animals. We recognize that the health status data from the current study should be interpreted with caution due to the relatively low number of affected animals and the high variability in health status of milk-diet calves during the transition period. However, the results obtained are supported by data on the live weight gains of sick dairy calves, which in the experimental group were 23% higher by the end of the recovery period.

### 3.2. Increase in Body Weight

**Figure 1:**
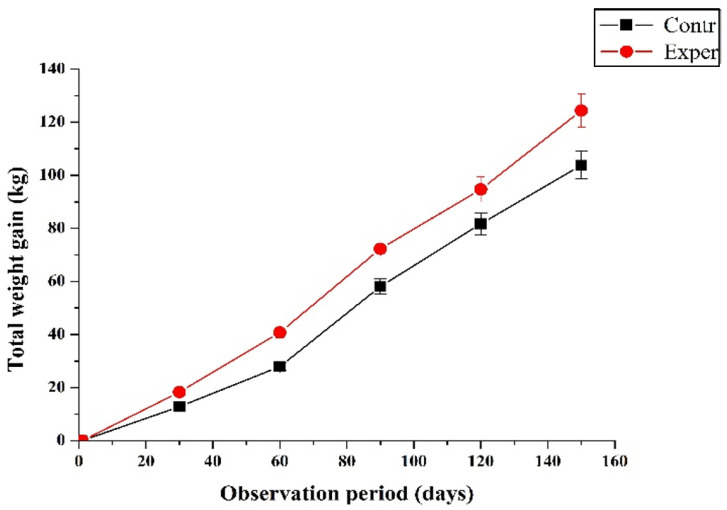
The effect of polyoctylated polysilicate hydrogel (POPSH) on the body weight gain of heifers (M ± m).

During the research, an increase in the gross body weight gain was observed, while significant differences (*p* = 0.046) were revealed between the groups at the end of the experiment (Figure 1).

When the growing heifer was exposed to stress factors associated with the regrouping of animals, the introduction of new ingredients into the diet of dairy calves (switching to a WMR and a solid diet at the end of the dairy period), we observed significant changes (*p* = 0.001) in the average daily live weight gain in the transition period.

The body weight gain increased in comparison with the control animals at the end of the first month of the experiment by 5.89 kg (7.37%); by the end of the milk period (60 days), by 14.43 kg (9.76%). By the end of the study (150 days), the calves of the experimental group surpassed the animals of the control in live weight by 22.0 kg, or 12.9%, and in total weight gain by 20.6 kg, or 19.8%.

At the same time, separate calculations showed that the consumption of digestible protein in the experimental group per unit of weight gain was reduced by 11.4% compared to the control group. Based on these facts, it can be assumed that the calves of the experimental group were less susceptible to the influence of a new environmental factor, in the form of a change in housing conditions and type of feeding, and more quickly adapted to it with less losses for the energy balance.

Similar results on the effect of feed adsorbents on calf weight gain were obtained when calves were fed hydrophilic adsorbents, for example, amorphous silicon dioxide [30], a mixture of peat homogenate and algae spirulina [31] and aluminosilicates: hongurin [32], glauconite [33] and clinoptilolite [34]. The closest, but still lower, results were demonstrated when studying the effect of peat suspension in combination with spirulina algae on the gain in live weight of calves aged 2 to 6 months, which in these experiments was 16.1% [31]. When using only algae in the same dose, but without peat, the increase was 8.41%. The authors, unfortunately, did not provide data on the use of only peat as an adsorbent but without spirulina [31]. For the rest of the mentioned adsorbents, the indicators were significantly lower. It should be noted here that it was precisely the adsorbents containing humic substances from peat, along with activated carbon but not aluminosilicates, that exhibited in vitro some activity against the lipophilic mycotoxin zearalenone [19]. These examples show that non-polar adsorbents appear to be more useful in protecting calves from feed toxins than their polar counterparts.

### 3.3. Blood Serum Biochemical Parameters

We assessed the course of metabolic processes in growing young animals during the dairy period of rearing by biochemical parameters of the blood serum (Table 3). There was a small change in some indicators, but all of them were within the physiological norm [35].

The concentration of total protein, albumin, and globulins was within the reference intervals, which indicates that the animals were in a healthy state and the study on feeding with POPSH did not have any negative effect on them. In animals of the experimental group, the protein index was 4.8% higher than the control values, but these changes did not have a statistically significant difference. The concentrations of Glu, Ca, P, Ca/P ratio, Chol, Fe, and TG in the blood serum of heifers from the control and experimental groups at the beginning and at the end of the experiment also did not differ significantly.

There was a tendency (*p* = 0.044) to increase the activity of the ALT enzyme, the indicator of which by the end of the milk period was 5.53 IU/L, or 28.8%, higher than in the heifers of the control group. The concentration of creatinine in the blood of heifers from the experimental group was also higher by 12.5 μmol/L, or 21.9%, compared with the control (*p* < 0.045). These data are consistent with the results of studies in which an increase in the activity of ALT and AST in calves and piglets, respectively, was observed against the background of the use of a feed adsorbent from glucomannans of cereal fiber [36,37]. The tendency for an increase in the protein index and an increased concentration of creatinine in the blood serum may indicate an intensification of protein metabolism in the experimental group. The issue of increased serum ALT concentration requires further study.

From the above, it follows that in the lactic and transitional phases of postnatal ontogenesis, when this transition requires physical and metabolic development of the rumen and coincides with the development of the salivary apparatus, the onset of rumination and significant physiological changes at the level of the intestine, liver and other tissues [1,38,39], the calves of the experimental group had more intensive metabolic processes in comparison with the control animals. This was manifested in their faster growth and development and confirmed by a significant increase in average daily weight gain at a lower relative cost of digested protein. This may indirectly indicate a decrease in the total toxic load from naturally contaminated feed under the action of POPSH, a decrease in energy consumption for metabolic neutralization of toxins, and protection of the digestive system from lipophilic toxins, and, in general, indicates a beneficial effect of the studied adsorbent on the health of heifers in the experimental context.

### 3.4. The Effect of POPSH on the Concentration of PCBs in the Blood of Heifers

It is known that PCBs are constantly present in feed for dairy cattle [2,5,7,40], accumulate in adipose tissue, are transferred to milk, have a negative effect on almost all the main functional systems of mammals and reduce their reproduction and productivity [28,41,42]. Moreover, as has been shown [43,44,45], PCBs can induce inflammatory processes in the intestine and disrupt intestinal permeability, microbiota, and metabolic homeostasis in general. According to the authors of another study, PCBs can influence markers of redox homeostasis in the blood of calves and participate in an increase in markers of inflammatory processes [40]. For this reason, reducing the load of these highly lipophilic ecotoxicants on productive animals is a very important task from the point of view of chemical protection of the internal environment of animals and humans.

Table 4 presents data on the concentrations of PCB congeners found in the blood of heifers from both groups at the end of the experiment and in the staple foods that were used in their diets. Data from single determinations for each type of samples are presented. Since single concentration measurements were carried out, those congeners are marked in the table (*), the difference in concentration of which in the blood of heifers was considered significant on the basis of the value of the measurement error. According to the laboratory data, the sensitivity of the applied analysis method was set as following: LOQ = 0.1 ng/g (limit of quantification); LOD = 0.02 ng/g (limit of detection); the error of the method in the determined concentration range is less than 22% (σ = ±22%) [46,47].

The used analysis protocol [26] makes it possible to quantify the sixty most frequently detected congeners from PCB#1 to PCB#209, including all indicator and all dioxin-like PCBs (DL-PCBs).

The table shows the data on the concentrations of PCB congeners in milk, WMR and blood of heifers, expressed in nanograms per 1 g of lipid fraction, and in the feed of the main diet, consisting of hay, haylage, silage and compound feed, in terms of dry weight. Until the end of the milk period (up to the 180th day after birth), the main amounts of PCBs enter the body of the heifer with milk and WMR. Since WMR is produced from skimmed milk, its contribution to the accumulation of PCBs in calves is usually estimated to be lower than from natural milk from the same farm [48]. From the data in Table 4, it can be concluded that milk is the most contaminated component in the diet of calves. However, when recalculating the concentration of PCBs in milk and WMR, not per 1 g of lipid, but per 1 g of the entire sample, it turns out that these components of the diet are arranged according to the degree of contamination in the following order: solid feed (7.4 ng/g), milk (1.11 ng/g), WMR (0.284 ng/g). Based on the data in the table, it can be assumed that congeners that are absent in the results of feed analysis are there at concentrations below LOQ (<0.1 ng/g) and are detected in the milk of lactating cows after their gradual and prolonged bioaccumulation in adipose tissue, with subsequent transfer into milk at higher concentrations.

The nomenclature of congeners in the blood of calves and milk is largely the same. We observed a strong positive correlation between the concentration of congeners in milk and in the blood of calves from the control group (R^2^ = 0.780). The correlation between milk and blood of animals from the experimental group was significant, but less pronounced (R^2^ = 0.686). It can be assumed that the main “load” of PCBs is accumulated by calves during the milk period and continues to increase after switching to solid feed. One may wonder how exactly changes in the level of PCBs in the blood can reflect their levels in other organs and tissues.

It has been established that, in humans, the level of some dioxin-like PCBs, polychlorinated dioxins and furans in blood serum, when referred to the lipid fraction, sufficiently reflects its concentration in the adipose tissues of the human body [49]. Similar results have been reported for the ratio of the concentrations of some OCPs in human blood and adipose tissue samples [50]. It is known that DDT and its main metabolites described in this work are very close to PCBs and dioxins in terms of distribution coefficients and other physicochemical properties. In other works, the authors noted a very strong degree of correlation (R^2^ = 0.9 ÷ 0.99) between the concentration level of indicator PCBs (138, 153, 180) in the blood serum and muscle tissues of sheep and cows [51,52]. A strong positive correlation (R^2^ = 0.789) was also found between the total concentration of indicator PCBs in the blood serum and meat of individual specimens (n = 56) of beef cattle delivered to the slaughterhouse [53]. A strong correlation was also noted between the content of indicator and dioxin-like PCBs in the adipose tissue of free-range goats, sheep, pigs, and cows and their content in the meat of the same animals on a fat basis [54]. Along with this, the authors noted the absence of a positive correlation between adipose tissue and liver. Based on the data on a high degree of correlation between the content of different PCBs in blood serum, adipose and muscle tissues, several research teams have proposed to conduct an in vivo assessment of the degree of contamination of animal meat with PCBs based on data on the level of their content in blood serum or fat [51,52,53,54,55].

These data suggest that, in calves, a decrease in the concentration of PCBs in the blood in a similar way reflects changes in their concentrations in adipose tissue and other compartments of the body, excluding liver, i.e., a decrease in the concentration of PCBs in blood samples in the experimental group, we believe, may reflect a decrease in the level of PCBs in the body of heifers as a whole. Here, it is necessary to make a small but fundamental reservation. Since in this study we did not directly measure the concentrations of PCBs in the adipose tissues of calves, then, despite the facts presented in the cited studies [49,50,51,52,53,54,55], we formally have no right to assert that POPSH decreases their concentrations not only in the blood, but also in other tissues of the calves. This study will be accomplished in the near future, and for now, for our part, we will restrict ourselves to conclusions at the level of assumptions.

**Figure 2:**
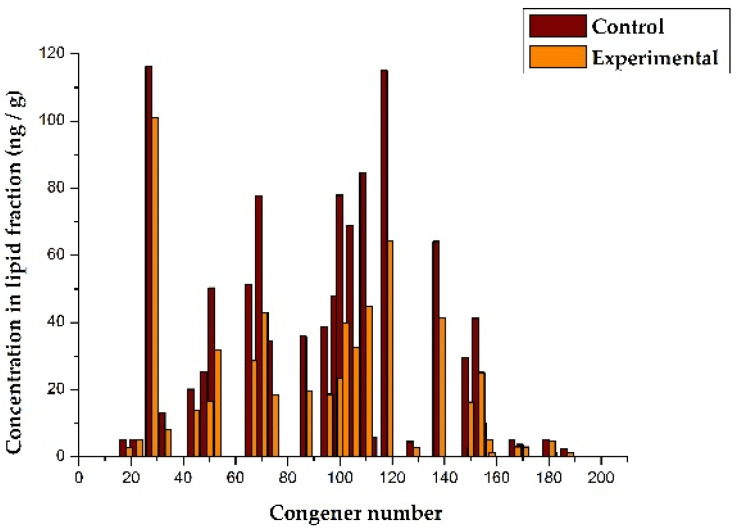
The effect of POPSH on the level of PCB congeners in blood of dairy heifers.

The data in Table 4 indicate that the total concentration of PCBs in the lipid fraction of the blood of calves from the experimental group (622 ng/g) is significantly lower than in calves from the control group (1038 ng/g). This may indicate that, as a result of the use of POPSH, the total load and pressure of PCBs on the physiological systems of the heifers of the experimental group decreased. Table 4 shows that, for 7 out of 33 congeners, the concentrations in the control and experimental groups differ insignificantly, but for the majority of congeners (n = 26) and for the total concentrations of PCB congeners in these groups, the differences are statistically significant at the *p* < 0.05 level. In Figure 2, the data on the concentrations of PCB congeners in the blood of calves are presented graphically.

**Figure 3:**
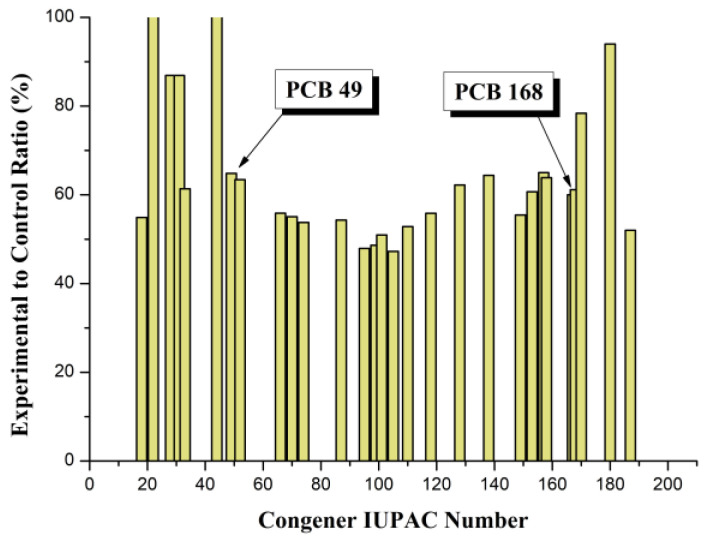
Influence of POPSH on the concentration ratios of PCB congeners.

The *X*-axis shows the congener numbers, according to the IUPAC nomenclature, which were determined in blood samples, and the *Y*-axis shows the concentrations of the corresponding congeners in the lipid fraction of the blood. It should be noted that different congeners vary in the degree to which their concentration decreases in the experimental group. In a separate graph, these differences are more noticeable. Figure 3 presents, in graphical form, the ratios of the concentrations of different congeners in the experimental group in relation to the control, expressed as a percentage.

In Figure 3, the X-axis is the same as in Figure 2, and the Y-axis shows the concentration of congeners in the experimental group relative to the control in percentages. A certain conditional range of congener numbers can be distinguished on the graph, in which the decrease in the concentration of congeners in the experimental group is most pronounced (35–52%) and statistically significant. This range, indicated by arrows in the figure, is located between numbers 49 and 168. It includes congeners containing 4 (PCB#49–PCB#74), 5 (PCB#87–PCB#118) and 6 (PCB#128–PCB#168) chlorine atoms. In the indicated range, the values of the partition coefficients of the detected PCB congeners in the octanol/water system (Log Pow) vary from 5.85 (PCB#49) to 7.27 (PCB#167) [28]. In our case, four out of seven indicator PCBs (52, 101, 138, 153), three out of four non-ortho (77, 81, 126) and seven out of eight mono-ortho dioxin-like PCBs (105, 114, 118, 123, 156, 157, 167) fall into this range. In other words, under the experimental conditions in the blood of calves, the concentration of those PCB congeners decreases to the greatest extent, among which are those most common substances in the environment (indicator PCBs) and the most toxic (dioxin-like PCBs) from this family of ecotoxicants. Interestingly, the most toxic dioxin-like congeners (PCB#126 and PCB#169) were not found in the samples studied. This is, to some extent, consistent with the results of the study, where it was shown that PCB congeners from the specified range (101, 118, 138, 149, 153) are also characterized by increased values of the transfer coefficients from feed to milk and, as a consequence, the coefficients of biomagnification in milk in lactating cows [56]. These observations require careful additional research.

It can be noted that this range of partition coefficients (Log Pow = 5.85 ÷ 7.27) includes many of the most toxic substances of other groups of hazardous ecotoxicants: PAHs, OCPs, polybrominated biphenyls, polybrominated diphenyl ethers, and chlorinated and brominated dibenzodioxins and dibenzofurans. Below, we will consider what well-known facts our discussion is based on. Table 5 shows the partition coefficients for PCB congeners from this range found in the blood of heifers and for some of the most toxic PCDDs and PCDFs.

It is known from the theory of reversed-phase (RP) liquid chromatography that the binding strength of any sorbate with a reversed-phase adsorbent in an aqueous medium is directly proportional to its partition coefficient (Log Pow) [57]. In this regard, a theoretical concept was developed of the influence of composition, structure and the presence of different structural elements of organic molecules on the values of partition coefficients [58,59]. These theoretical premises were fully confirmed in practice by using reversed-phase high-performance liquid chromatography (RP–HPLC) to determine partition coefficients [60,61,62]. In these studies, on a practical level, the presence of a direct dependence of the binding strength of the sorbate with the RP-adsorbent from the value of the sorbate’s partition coefficient was confirmed. Currently, this RP–HPLC application is a convenient alternative or addition to the classical shake-flask method [63] for determining the partition coefficients of moderately lipophilic and lipophilic substances. Taking into account the fact that POPSH is a typical RP-adsorbent on a polysilicate base, and the most toxic tetra-, penta- and hexachlorodibenzodioxins and dibenzofurans in terms of their partition coefficients fall within the indicated range [29], it is possible to predict with a high degree of confidence that all these toxins will bind to POPSH and be eliminated from the digestive tract with an efficiency comparable, at least, to the studied PCBs.

Over the past 40 years, a large number of articles and reviews devoted to the problems of removing various POPs from animal or human organisms have been published in the scientific literature [64]. For these purposes, a polycationic adsorbent based on porous polystyrene—cholestyramine [65], activated carbon [66,67,68,69], nonabsorbable lipid-like sucrose esters [70,71,72] was used to accelerate the elimination of these substances from the body. Barbiturates were also used to accelerate the metabolism of POPs in the liver and to remove toxins and their metabolites from the body [69,73]. In most cases, the effectiveness of such attempts was not as high as expected in terms of safety parameters and agricultural applicability from the point of view of economic viability and, at present, such methods have not found wide application in practical agriculture. The use of POPSH against the background of the presented data looks at least quite comparable, if not better, in terms of efficiency and economic feasibility of its use in beef and dairy cattle breeding.

There is also one more approach described recently [19,48]. Fattening beef cattle whose level of PCBs in their tissues exceeded permissible normalized values were transported to remote farms and kept there in a more “clean” environment and on cleaner feed until the level of PCBs decreased and values acceptable by law were reached. Such decontamination occurs, according to the authors, due to the elimination of the source of intoxication from the feed, metabolic transformations and the elimination of a portion of the accumulated PCBs and their metabolites from the body naturally with urine and feces, as well as a decrease in the concentration of the remaining part of the accumulated toxins due to physical dilution with an increase in weight. An alternative or addition to this practice is, in our opinion, the use of feed adsorbents that can reduce the level of toxins in blood and tissues, even when using contaminated feed, which was demonstrated in this work. It is possible that the simultaneous joint use of quarantine in “clean” conditions with the use of “clean” feed and effective non-polar adsorbents can improve decontamination efficiency and reduce the quarantine time and the corresponding costs.

On the basis only of the data obtained, it is still difficult to draw a reasonable conclusion about the reasons for the uneven decrease in the concentration of various congeners in the blood of calves. Many biochemical [74,75] and physicochemical [13] processes at the organ and interstitial levels, which are still not well understood, are involved in the implementation of the processes of accumulation and elimination of lipophilic xenobiotics from the body.

There are two main routes of entry of PCBs into the digestive tract of mammals. The first is the direct ingestion of PCBs with contaminated food. The second way is derived from enterohepatic circulation (EHC), in which lipophilic xenobiotics and their metabolites, previously accumulated in adipose tissue, enter the duodenal lumen from the gallbladder together with bile acids during food digestion [76,77]. So far, we do not have direct evidence of the involvement of POPSH in decontamination from PCBs in both processes. At the same time, there are data on the use of adsorbents and other pharmacological agents capable of opening the hepato-enteric loop to accelerate the elimination of lipophilic xenobiotics from the body [77,78]. Based on the totality of these data, it can be assumed with caution that POPSH is not only able to protect the digestive tract and other internal organs from PCBs supplied with food and prevent their accumulation, but also, probably, to interrupt the EHC of these POPs, gradually reducing their total concentration in adipose tissue and in the body as a whole.

The question of the rate-limiting parameters of the decontamination process through EHC remains open. Whether the rate of elimination depends on the applied dose of the adsorbent, or whether this process is limited by the volume of hepato-enteric circulation after feed intake, is not entirely clear. This issue requires multilateral studies to improve the efficiency of decontamination with the help of feed adsorbents. It should be borne in mind that, in addition to PCBs, feed for dairy cattle, as mentioned above, always contains other POPs, mycotoxins, PAHs and other toxic impurities. Consequently, the additional weight gain obtained in the experimental group of heifers serves as a kind of integral indicator that reflects the overall degree of reduction in the level of intoxication of animals with the entire set of lipophilic toxins from feed, including, probably, PAHs, other POPs and mycotoxins, under the influence of a non-polar adsorbent. All this requires additional careful study.

These circumstances should be taken into account when calculating the doses used. In the case of using POPSH as an adsorbent only to protect the digestive tract of animals from the damaging effect of POPs and other lipophilic toxins from feed and prevent their subsequent bioaccumulation, relatively low doses of the adsorbent (0.05–0.2 g/kg live weight) are likely to be enough. For decontamination of growing young stock and lactating cows, the dose is likely to be higher and can only be determined empirically, taking into account the level of feed contamination, the concentration of toxins in the blood and milk, and the desired degree of decontamination of livestock and products. This requires additional research in the field of pharmacokinetics and pharmacodynamics of the accumulation and excretion of PCBs and other POPs. Possibly, with the proper choice of doses, modes of application and duration of decontamination, the result will be basic livestock products with reduced levels of contamination or entirely free of lipophilic toxins. These goals and objectives form the further directions of our research.

## 4. Conclusions

This study, for the first time, demonstrates the possibility of reducing the concentrations of different PCB congeners in the blood of dairy heifers using a non-polar feed adsorbent. This indicates that POPSH, by binding PCBs in the digestive tract, helps to protect the digestive tract and other organs and to reduce the concentration of PCBs and, probably, other lipophilic toxins in the blood of heifers, thus reducing the overall toxic load on the whole body from the naturally contaminated feed used, and performing the function of decontamination of animals. This is expressed in a more dynamic weight gain, a decrease in specific feed consumption and leads to a better realization of the productive potential of growing young cattle.

## Figures and Tables

**Table 1:** Structure of the heifer’s solid diet based on dry matter.

Type of Feed	Content in Total Diet (%)
Roughage	4.8
Concentrated feed	57.4
Juicy feed	37.8

**Table 2:** The incidence of diseases in heifers and their impact on productivity.

Index	Group C (n = 19)	Group E (n = 20)
Diseases of the digestive system, n	3	3
Respiratory tract diseases, n	1	-
Average daily weight gain of patients for the treatment period, g	483.3 ± 43.7	597.2 ± 51.4 *
Average daily weight gain of patients for the total period, g	573.6 ± 61.3	715.5 ± 65.2 *

*—Significant at *p* < 0.05.

**Table 3:** Biochemical and morphological parameters of the heifers’ blood (M ± m, n = 5).

Parameter	Group	
	Control	Experimental
Total protein, g/L	73.5 ± 1.60	73.2 ± 2.28
Albumin, g/L	33.6 ± 0.75	33.8 ± 0.40
Globulin, g/L	39.8 ± 1.14	39.45 ± 1.20
A/H ratio	0.82	0.86
ALT, IU/L	19.2 ± 2.46	24.7 ± 0.86 *
AST, IU/L	68.5 ± 3.21	68.6 ± 2.17
Alkaline phosphatase, IU/L	420.0 ± 31.76	413.8 ± 53.32
Glucose, mmol/L	6.49 ± 0.20	6.15 ± 0.60
Calcium, mmol/L	2.82 ± 0.06	2.82 ± 0.04
Phosphorus, mmol/L	3.23 ± 0.15	3.36 ± 0.19
Ca/P ratio	1.14 ± 0.07	1.10 ± 0.07
Creatinine, μmol/L	57.0 ± 1.66	69.5 ± 5.77 *
Cholesterol, mmol/L	3.37 ± 0.39	3.75 ± 0.29
Bilirubin, μmol/L	5.24 ± 0.96	4.85 ± 1.55
Iron, μmol/L	27.0 ± 2.20	28.1 ± 0.99
Triglycerides, mmol/L	0.32 ± 0.02	0.30 ± 0.02
Leukocytes, 10^9^/L	11.9 ± 1.33	12.0 ± 1.32
Erythrocytes, 10^12^/L	12.3 ± 0.39	11.5 ± 0.92
Hemoglobin, g/L	115.0 ± 3.37	106.8 ± 4.39
Hematocrit, %	45.1 ± 0.82	33.7 ± 9.97

*—Significant at *p* < 0.05.

**Table 4:** Concentration of PCBs in feed components and in heifers’ blood.

IUPAC Congener Number	Milk (ng/g of Lipid)	WMR (ng/g of Lipid)	Solid Feed (ng/g of DM *)	Blood (ng/g of Lipid)	
				Contr.	Exper.
18	0.35	<0.1	<0.1	5.1	2.8 *
19	<0.1	0.2	<0.1	<0.1	<0.1
22	0.2	<0.1	<0.1	5.1	5.1
28 ^a^	0.75	0.8	0.15	58.1	50.5
31	0.75	0.8	0.15	58.1	50.5
33	0.33	<0.1	<0.1	13.2	8.1 *
37	0.2	<0.1	<0.1	<0.1	<0.1
44	0.33	0.4	0.2	13.8	20.1 *
49	0.4	1.0	0.1	25.3	16.4
52 ^a^	0.8	2.3	0.2	50.3	31.9 *
66	1.4	0.9	0.4	51.2	28.6 *
70	1.6	1.4	0.4	77.7	42.8 *
74	1.4	0.6	0.3	34.4	18.5 *
87	0.9	0.4	0.2	35.9	19.5 *
95	0.5	0.7	0.2	38.8	18.6 *
99	3.3	1.0	0.5	47.9	23.3 *
101 ^a^	1.3	1.1	0.8	77.9	39.7 *
105 ^b^	2.1	0.5	<0.1	68.8	32.5 *
110	1.5	0.9	0.7	84.6	44.7 *
114 ^b^	0.15	<0.1	<0.1	5.8	<0.1 *
118 ^b^	5.1	1.0	1.1	115.0	64.2 *
119	0.1	<0.1	<0.1	<0.1	<0.1
128	0.3	<0.1	<0.1	4.5	2.8 *
138 ^a^	1.35	0.4	0.1	32.0	20.6 *
149	0.5	0.3	0.2	29.4	16.3 *
151	0.1	<0.1	<0.1	2.5	<0.1 *
153 ^a^	0.9	0.2	0.5	20.6	12.5 *
156 ^b^	0.4	0.1	<0.1	9.9	5.0 *
157 ^b^	0.1	<0.1	<0.1	2.0	1.3
158	1.35	0.4	0.2	32.1	20.5 *
167 ^b^	0.2	<0.1	<0.1	5.0	3.0 *
168	0.9	0.2	<0.1	20.6	12.6 *
170	0.2	<0.1	0.3	3.7	2.9
177	0.1	<0.1	<0.1	<0.1	<0.1
180 ^a^	0.3	0.1	0.7	5.0	4.7
183	0.1	<0.1	<0.1	1.1	<0.1 *
187	0.1	n.d.	0.1	2.5	1.3 *
Fat content (%)	3.69	14.46	<0.1	0.17	0.19
Total (ng/g)	30.0	15.7	7.4	1038	622 *

DM—dry matter; *—differences are statistically significant (*p* < 0.05; σ = ±22%); ^a^—indicator PCBs; ^b^—DL-PCBs.

**Table 5:** Partition coefficients of some congeners of PCBs, and polychlorinated dioxins and furans.

Compound (PCB#)	Log Pow *	Compound (PCB#)	Log Pow *	Compound	Log Pow **
44	5.75	118 ^b^	6.74	PCDD	
49	5.85	119	6.58	2,3,7,8-TCDD	6.80
52 ^a^	5.84	128	6.74	1,2,3,7,8-PeCDD	6.64
66	6.20	138 ^a^	6.83	1,2,3,4,7,8-HxCDD	7.80
70	6.20	149	6.67	PCDF	
74	6.20	151	6.64	2,3,7,8-TCDF	6.53
87	6.29	153 ^a^	6.92	1,2,3,7,8-PeCDF	6.79
95	6.13	156 ^b^	7.18	2,3,4,7,8-PeCDF	6.92
99	6.39	157 ^b^	7.18		
101 ^a^	6.38	158	7.02		
105 ^b^	6.65	167 ^b^	7.27		
110	6.48	168	7.11		
114 ^b^	6.65				

*—data from [28]; **—data from [29]; ^a^—indicator PCBs; ^b^—DL-PCBs.

## Data Availability

All data are contained in the manuscript.
